# The Effects of Neonatal Zingerone Administration and Adolescent Alcohol Exposure on Bone Health Markers and Morphometry in Male Sprague–Dawley Rats

**DOI:** 10.1155/bmri/7060593

**Published:** 2026-04-29

**Authors:** Brunhildé De Vos, Bernice Asiedu, Anna M. Joubert, Abe E. Kasonga, Trevor T. Nyakudya

**Affiliations:** ^1^ Department of Human Physiology, School of Medicine, Faculty of Health Sciences, University of Pretoria, Pretoria, South Africa, up.ac.za

**Keywords:** alcohol consumption, bone health, bone microarchitecture, zingerone

## Abstract

**Objective:**

Neonatal interventions influence long‐term developmental outcomes, with excessive alcohol consumption impairing metabolism and bone health. Zingerone, a natural antioxidant, may possess health benefits against alcohol‐induced oxidative damage and inflammation, promoting bone health. This study investigates the effects of zingerone on markers of bone turnover and morphometry in neonatal rats subjected to alcohol exposure during critical developmental stages.

**Methods:**

Ten‐day‐old male Sprague–Dawley rats (*N* = 35) were randomized and treated with water (*C*), zingerone (*Z*) (40 mg/kg), alcohol (*A*) (1 g/kg) or zingerone–alcohol combination (ZA) for 9 days. During adolescence, rats received either water or a secondary A insult (20% *v*/*v*) for 54 days. At termination, ELISA kits were used to measure biomarkers of bone formation (bone‐specific alkaline phosphatase, osteocalcin, and procollagen Type I N‐terminal propeptide). Bone morphology and morphometry was assessed using microcomputed tomography.

**Results:**

Neonatal zingerone (*Z* + *A*; *Z* *A* + *A*) and double alcohol exposure (*A* + *A*) significantly increased plasma BALP and P1NP levels (*p* < 0.05). Tibial length remained unchanged across groups (*p* > 0.05). Neonatal zingerone with adolescent alcohol (*Z* + *A*) significantly reduced tibial mass, bone mass‐to‐length ratios, and trabecular architecture near the growth plate (*p* < 0.05). Neonatal and adolescent administration of alcohol (*A* + *A*) significantly increased midshaft cortical thickness (*p* < 0.05).

**Conclusion:**

Adolescent alcohol treatment impaired bone function and morphology, neonatal zingerone alone offering limited protection. Coadministration of zingerone with alcohol neonatally produced variable and context‐dependent changes with no clear evidence of sustained protection against the effects of alcohol exposure. These findings underscore the importance of optimizing timing, dosage, and combination therapies for bone health. Further studies are warranted to clarify whether zingerone has clinically meaningful bone‐protective effects.

Impact Statement

Bone health is crucial for overall well‐being, yet excessive alcohol consumption during early development can lead to lasting skeletal impairments. This study investigates the potential of zingerone, a bioactive compound from ginger, to mitigate alcohol‐induced bone deterioration. By examining its effects on bone structure and biochemical markers in a preclinical model, our findings highlight the complexities of alcohol’s impact on bone and explore zingerone’s potential as a protective agent. This research paves the way for future studies on dietary interventions to support bone health and prevent metabolic bone disorders linked to early‐life alcohol exposure.

## 1. Introduction

Excessive alcohol consumption presents a significant public health concern, particularly during critical developmental stages such as pregnancy, lactation, and adolescence [1]. Several studies have shown that maternal alcohol intake during breastfeeding negatively impacts offspring health, increasing the risk of metabolic disorders like hepatic steatosis, obesity, and insulin resistance later on in adulthood [2, 3]. Recent research increasingly demonstrates that alcohol consumption during adolescence, a period marked by rapid growth and metabolic development, can disrupt critical stages of brain maturation, elevate the risk of substance dependence, and lead to poor health and social outcomes [4]. Although postnatal alcohol exposure is harmful at any stage, it is specifically detrimental during the suckling and adolescent periods. Neonates and adolescents are especially vulnerable to the long‐term metabolic consequences of early‐life alcohol exposure, potentially leading to lasting disruptions in metabolic homeostasis, including bone metabolism and health [5].

Most studies investigating the effects of alcohol on metabolism have primarily focused on gestation and adulthood [6]. Few studies have assessed the effects of alcohol consumption during both the neonatal stage and adolescence. This gap in research is particularly important because alcohol exposure at these stages can have distinct and lasting consequences on metabolic health and bone development later on in adulthood [6]. In light of the risks associated with alcohol consumption on bone metabolism during critical developmental periods, there is growing interest directed towards interventions aimed at mitigating these harmful effects. Studies have also shown that dietary interventions with medicinal plant extracts and phytochemicals during the neonatal phase may promote favorable long‐term health outcomes by counteracting the early metabolic disruptions caused by alcohol exposure [7]. Zingerone, a bioactive phytochemical compound derived from cooked ginger (*Zingiber officinale*), was selected for this study due to its health benefits attributed to its antioxidant and anti‐inflammatory properties [8].

Given these therapeutic attributes, we hypothesize that zingerone may offer beneficial effects against alcohol‐induced changes in the bone, potentially mitigating the deterioration of bone health associated with alcohol exposure during critical periods of developmental plasticity. This study is aimed at investigating the effect of neonatal zingerone administration on bone health in a rodent model of alcohol‐induced changes in the bone, providing insights into its potential benefits in maintaining bone health. Bone‐specific alkaline phosphatase (BALP), osteocalcin (OC), and procollagen Type I N‐terminal propeptide (P1NP) are established surrogate biomarkers of bone formation and turnover, reflecting osteoblast activity, bone matrix mineralization, and Type I collagen synthesis, respectively. Previous studies report that chronic high‐dose alcohol exposure can suppress these markers by impairing osteoblast function, whereas acute or low‐dose exposure may produce transient increases as part of a compensatory bone remodeling response [9, 10].

## 2. Materials and Methods

### 2.1. Ethical Clearance and Animal Housing

All experimental animal treatments were conducted at the University of the Witwatersrand (WITS), Research Animal Facility (WRAF), Johannesburg, South Africa as part of an ongoing project research study by Asiedu (2023). Ethical approval was granted by the WITS Animal Research Ethics Committee (Ethical Clearance No. 2019/10/57/B), with additional approval for sample handling and testing from the University of Pretoria (UP) Animal Ethics Committee, South Africa (Ethical Clearance No. 491/2022). The study complied with the South African National Standard (SANS 10386:2008) in accordance with ethical animal use and the Animals Protection Act 1962.

Ten‐day‐old male Sprague–Dawley rat pups (*N* = 35) were used for experimental treatments. The pups were sourced from litters of primiparous dams and culled to litter sizes of 8–12 pups per litter as described by Suvorov et al. [11]. Prior to weaning, the pups were housed with their dams in acrylic cages, while postweaning, they were individually housed in separate acrylic cages containing wood shavings as bedding. Environmental conditions were maintained within the thermoneutral zone, with an ambient temperature of 24°C±1°C and relative humidity of 55*%* ± 10*%*. A 12 h light–dark cycle was strictly followed (with lights on at 07:00). The rats were acclimated for 2 days prior to the initiation of experimental treatments. Throughout the study, the animals had ad libitum access to both standard rodent chow (Epol, Johannesburg, South Africa) and water, in addition to the experimental interventions.

### 2.2. Reagent Preparation

All neonatal experimental groups received nutritive rat milk (Kyron kitty milk, Kyron Labs, Johannesburg, South Africa) as the vehicle, adjusted to an oral administration volume of 10 mL/kg [12]. Ethanol dosage was determined by the body surface area (BSA) normalization method, following the human‐to‐rat conversion ratio of 1:6.17, as outlined by Reagan‐Shaw et al. [13]. Therefore, based on the BSA calculation, rat pups received ethanol, prepared in nutritive milk, at a dose of 1 g/kg of body mass per day.

### 2.3. Preparation of Zingerone

Zingerone (C_11_H_14_O_3_, 194.23 g/mol) was purchased from Sigma‐Aldrich Inc. (W312401, Johannesburg, South Africa). Neonatally orally administered zingerone was prepared in nutritive milk at a dose of 40 mg/kg of body mass. This dosage was deemed safe and effective for rat pups, consistent with findings from Muhammad et al. [12].

### 2.4. Study Design

**Figure 1:**
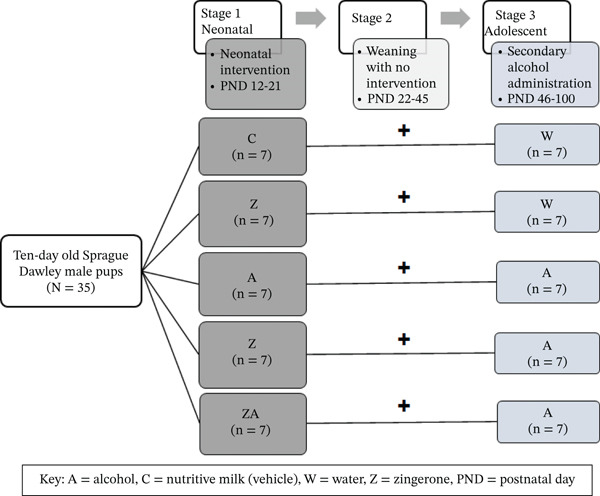
Schematic diagram of the experimental conditions following neonatal and adolescent intervention. The study was divided into three stages of interventions. Stage 1, the neonatal rat pups were subjected to treatments (PND 12–21). Stage 2, no treatments were provided during this phase (PND 21–46). Stage 3, the adolescent rats received a second alcohol hit. Group 1 (*C*) received no treatment in the neonatal phase and during adolescence; Group 2 (*Z* + *W*) was treated with zingerone (40 mg/kg) in the neonatal phase and water during adolescence; Group 3 (*A* + *A*) treated with alcohol during both the neonatal and adolescent phase (1 g/kg ethanol); Group 4 (*Z* + *A*) treated with zingerone in the neonatal phase and alcohol in adolescence; Group 5 (*Z* *A* + *A*) treated with a combination of zingerone‐alcohol in the neonatal phase, followed by alcohol in adolescence. PND—postnatal day; *A*—alcohol; *C*—control; *W*—water; *Z*—zingerone; ZA—zingerone‐alcohol; *n* = 7 per treatment group.

The experiment period consisted of three distinct phases (Figure 1) as described by Asiedu et al. [14]: Phase 1—neonatal intervention (postnatal Days [PND] 12–21), Phase 2—weaning with no intervention (PND 22–45), and Phase 3—secondary alcohol administration (PND 46–100). During the first phase, rats were randomly divided into five groups based on Developmental Origins of Health and Disease (DOHaD) principles, with treatments administered via oral gavage [15].

1. Group 1: (*C*)—rats were orally gavaged with nutritive milk during the neonatal phase and received ad libitum access to drinking water during adolescence, *n* = 7.
2. Group 2: (*Z* + *W*)—rats were orally gavaged with zingerone (40 mg/kg body mass) during the neonatal phase and received ad libitum access to drinking water during adolescence, *n* = 7.
3. Group 3: (*A* + *A*)—rats were treated with a double hit of alcohol during the neonatal phase (1 g/kg body mass alcohol) and received ad libitum access to drinking water containing 20% alcohol (*v*/*v*) during adolescence, *n* = 7.
4. Group 4: (*Z* + *A*)—rats were orally gavaged with zingerone (40 mg/kg body mass) during the neonatal phase and received ad libitum access to drinking water containing 20% alcohol (*v*/*v*) during adolescence, *n* = 7.
5. Group 5: (ZA + *A*)—rats were orally gavaged with a combination of zingerone (40 mg/kg body mass) and alcohol (1 g/kg of body mass) during the neonatal period, followed by ad libitum access to drinking water containing 20% alcohol (*v*/*v*) during adolescence, *n* = 7.

In the second phase, no interventions were applied, and rat pups were allowed to mature into adolescence with ad libitum access to standard rat chow and tap water. In the third phase, during adolescence (PND 46–100), a second hit of alcohol (*A*) or tap water (*W*) was introduced as drinking fluids ad libitum for 8 weeks. Alcohol‐fed adult rats (*Z* + *A*, *A* + *A*, and *Z* *A* + *A*) were given tap water containing alcohol which was progressively increased from PND 46: 5% (*v*/*v* alcohol) (PND 46–52), 10% (*v*/*v*) (PND 53–59), to 20% (*v*/*v*) (PND 60–100) [16]. Incremental alcohol exposure increased the success rate of alcohol‐induced metabolic and bone dysfunction by impairing lipid metabolism and elevating oxidative stress in adolescent rats [14].

### 2.5. Terminal Procedure and Sample Collection

Terminal procedure was performed at WITS according to the experimental protocol by Asiedu et al. In brief, at the end of the 100‐day treatment period, rats were fasted overnight (12 h) and euthanized on Day 101 via intraperitoneal overdose injection of sodium pentobarbital (200 mg/kg; Euthanase, Bayer, Johannesburg, South Africa). Blood was collected via cardiac puncture into heparinised tubes, centrifuged at 3000 × g for 15 min, and plasma samples were stored at −80°C for enzyme‐linked immunosorbent assay (ELISA) quantification of bone markers. The right tibia was dissected, cleaned, dried for 5 days at 60°C, weighed (Presica 310M; Presica Instruments AG, Dietikon, Switzerland), and stored at room temperature in a desiccator for bone morphometry analysis [14]. Samples were exclusively collected from male rats to mitigate physiological interferences associated with the female hormonal cycle.

### 2.6. Measurements

#### 2.6.1. Biochemical Analysis of Surrogate Markers of Bone Health and Metabolism

The plasma activity of BALP (Catalog No. ER0761, FineTest, Wuhan, China), OC (Catalog No. ER1205, FineTest, Wuhan, China), and P1NP (Catalog No. E‐EL‐R1414, Elabscience, Houston, Texas, United States) were determined in rat plasma as described by the manufacturer′s instructions. Optical density (OD) was measured at 450 and 600 nm using an epoch microplate spectrophotometer (BioTek, Winooski, Vermont, United States) [17]. Plasma concentrations of BALP, OC, and P1NP were calculated using the standard curves generated by their irrespective OD′s.

#### 2.6.2. Tibial Morphological Measurements: Longitudinal Growth

Longitudinal bone growth was assessed using the right tibiae (*N* = 35). Dry bone mass (mg) was recorded using a Presica 310M electronic scale (Presica Instruments AG, Dietikon, Switzerland). The length (mm) of each tibia was measured from the tibia head to the medial malleolus [18]. Additionally, bone mass‐to‐length ratio (BMLR) (mg/mm) was determined as an indication of bone density per unit of length, a metric commonly used to assess bone strength and health, calculated using Equation (1) below [18].

(1) Bone−mass−to−length ratio mg/mm=mass of tibia mglength of tibia mm

#### 2.6.3. Microcomputed Tomography (Micro‐CT) Analysis

In the current study, relative bone density and microarchitecture of the tibiae (*N* = 35) were evaluated at South African Nuclear Energy Corporation (NECSA) using nondestructive three‐dimensional micro‐CT imaging (Nikon X‐TEK XT H 225 L METRIS, Tring, United Kingdom) with guidelines from Kim et al. [19] and Bouxsein et al. [20] (Supporting Information, Figure S1). Tibiae were vertically positioned in florist foam, with a rubber‐like adhesive (i.e. Prestik; Bostik, South Africa) as a reference marker, allowing 3–4 bones to be scanned at a voxel resolution of 14 *μ*m or 7 bones at 29 *μ*m. Imaging parameters were set at 100 kV and 100 *μ*A with 1000 projections and an exposure time of 2000 ms (Figure S1). Reconstructed 3D images were processed using Nikon CT Pro 3D and analyzed with VGStudio MAX 2022.4 software (Volume Graphics GmbH, Heidelberg, Germany). Trabecular microarchitecture was assessed in the proximal tibial metaphysis at 14‐*μ*m resolution, starting 1 mm away from the growth plate (Supporting Information, Figure S2; indicated by the red line) extending 3 mm longitudinally. Although cortical bone thickness was evaluated at the tibial midshaft using 29‐*μ*m resolution [21]. A 1‐mm cross‐sectional slice was extracted from the midshaft and cortical bone was quantified using the plug‐in tool “radial_thickness_measure.jar” (designed by Robert Nshimirimana, NECSA, South Africa). Trabecular parameters such as trabecular bone volume fraction (BV/TV, %), bone surface density (BS/BV, mm^−1^), trabecular thickness (Tb.Th, mm), trabecular separation (Tb.Sp, mm), and trabecular number (Tb.N, mm^−1^), were evaluated as indicators of relative bone mineral density (BMD) [22]. Cortical thickness (Ct.Th) was quantified via ImageJ software (National Institutes of Health, Version 1.53r).

### 2.7. Statistical Analysis

Data was presented as mean ± standard deviation with a minimum of three samples per treatment group. Statistical analysis was performed using GraphPad Prism 9 (San Diego, California, United States) using one‐way ANOVA with Bonferroni′s multiple comparisons as a post hoc test. Statistical significance was set at *p* ≤ 0.05.

## 3. Results

### 3.1. Effects of Neonatally Administered Zingerone and Adolescent Alcohol Exposure on Plasma Markers of Bone Turnover

**Figure 2:**
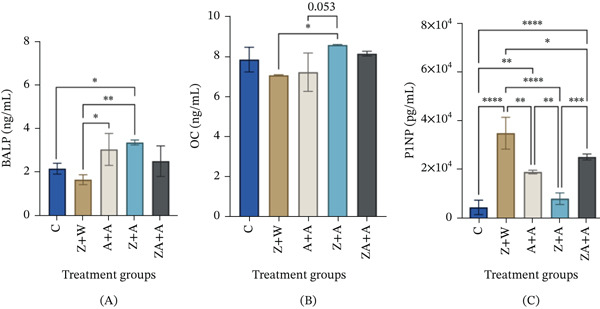
Effects of neonatally administered zingerone (40 mg/kg/day) and adolescent alcohol (1 g/kg) on plasma concentrations of (A) BALP, (B) OC, and (C) P1NP as bone health markers. Data were presented as mean ± standard deviation. BALP, OC, and P1NP levels were elevated in the alcohol‐treated groups with or without zingerone. Statistically significant if  ^∗^ *p* ≤ 0.05,  ^∗∗^ *p* ≤ 0.01,  ^∗∗∗^ *p* ≤ 0.001,  ^∗∗∗∗^ *p* ≤ 0.0001 between treatment groups. Group 1 (*C*) received no treatment in the neonatal phase and during adolescence; Group 2 (*Z* + *W*) was treated with zingerone (40 mg/kg) in the neonatal phase and water during adolescence; Group 3 (*A* + *A*) treated with alcohol during both the neonatal and adolescent phase (1 g/kg ethanol); Group 4 (*Z* + *A*) treated with zingerone in the neonatal phase and alcohol in adolescence; Group 5 (ZA + *A*) treated with a combination of zingerone–alcohol in the neonatal phase, followed by alcohol in adolescence. *A*—alcohol; *C*—control; *W*—water; *Z*—zingerone; ZA—zingerone‐alcohol; BALP—bone‐specific alkaline phosphatase, OC—osteocalcin, P1NP—procollagen Type 1 N‐terminal propeptide.

Neonatal administration of zingerone followed by alcohol administration during adolescence (*Z* + *A*) caused a significant increase in the plasma of BALP compared with the control group (Figure 2A, *p* = 0.0489). Plasma BALP was significantly increased in rats that administered a double hit of alcohol neonatally and in adolescence (*A* + *A*) compared with the *Z* + *W* group (*p* = 0.0344). There was a nonsignificant increase in the plasma levels of BALP in ZA + *A* compared with the control (*p* = 0.9009). Neonatal treatment with zingerone followed by alcohol treatment in adolescence (*Z* + *A*) significantly increased OC levels compared with the *Z* + *W* group (Figure 2B, *p* = 0.05). Neonatal zingerone treatment with no intervention during adolescence (*Z* + *W*) resulted in a significant increase in P1NP levels compared with other treatment groups (Figure 2C, *p* < 0.05). Neonatal and adolescent administration of alcohol with neonatal zingerone (ZA + *A*) and without neonatal zingerone (*A* + *A*) caused a significant increase in P1NP levels when compared with the control (Figure 2C; *p* < 0.0001). The combined administration of neonatal zingerone‐alcohol followed by alcohol administration in adolescence (ZA + *A*) had no effect on plasma BALP or OC levels (Figure 2A,B; *p* > 0.99). However, ZA + *A* significantly increased P1NP levels when compared with the control group (Figure 2; *p* < 0.0001).

### 3.2. Determination of Bone Morphometry

#### 3.2.1. Effect of Neonatally Administered Zingerone and Adolescent Alcohol Exposure on Tibial Mass, Length, and BMLR Ratio

**Figure 3:**
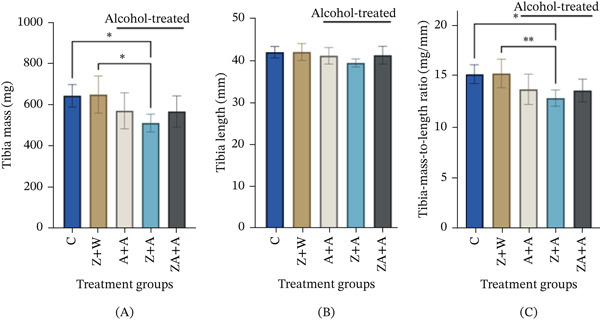
The impact of neonatally administered zingerone (40 mg/kg) and adolescent alcohol (1 g/kg) on tibia mass, length and tibia mass‐to‐length ratio in actively growing Sprague–Dawley male rats. Data were presented as mean ± standard deviation and considered statistically significant if  ^∗^ *p* ≤ 0.05,  ^∗∗^ *p* ≤ 0.01 (*n* = 7 per group). Group 1 (*C*) received no treatment in the neonatal phase and during adolescence; Group 2 (*Z* + *W*) was treated with zingerone (40 mg/kg) in the neonatal phase and water during adolescence; Group 3 (*A* + *A*) was treated with alcohol during both the neonatal and adolescent phases (1 g/kg ethanol); Group 4 (*Z* + *A*) was treated with zingerone in the neonatal phase and alcohol in adolescence; Group 5 (ZA + *A*) was treated with a combination of zingerone–alcohol in the neonatal phase, followed by alcohol in adolescence. *A*—alcohol; *C*—control; *W*—water; *Z*—zingerone; ZA—zingerone‐alcohol; *n* = 6 − 7 per treatment group.

Neonatal zingerone administration and alcohol later on in adolescence (*Z* + *A*) significantly decreased tibial mass when compared with the control (Figure 3A; *p* = 0.0226). There were no significant changes in tibial bone length across all treatment groups (*p* > 0.05; Figure 3B). Neonatal orally administered zingerone with alcohol during adolescence (*Z* + *A*) resulted in a significant decrease in bone‐ BMLR compared with the control (Figure 3A,C; *p* = 0.0099). There was a nonsignificant decrease in BMLR of all the alcohol‐treated groups (*A* + *A* and ZA + *A*) when compared with the control (Figure 3C; *p* > 0.05).

#### 3.2.2. Effect of Neonatal Administered Zingerone and Adolescent Alcohol Exposure on Internal Skeletal Morphometry (Trabecular and Cortical Bone) via Micro‐CT Parameters

Neonatal and adolescent administration with alcohol (*A* + *A*) did not affect the trabecular bone morphometry at the proximal tibial metaphysis region when compared with the control group (*p* > 0.05; Table 1). However, neonatal administration with zingerone followed by the adolescent intervention with alcohol (*Z* + *A*) led to a significant decrease in the quality of the trabeculae near the tibial growth plate, as indicated by a significant increase in the bone surface density (BS/BV, *p* = 0.0133) and trabeculae number (Tb.N, *p* = 0.0133) with decreased trabecular thickness (Tb.Th, *p* = 0.0183) when compared with the control (Table 1). Similar structural deteriorations were observed in the *Z* + *W* group, which received zingerone exclusively during the neonatal phase, showing a significant increase in BS/BV (*p* = 0.0394) with a significant decrease in bone volume fraction (BV/TV, *p* = 0.0089), trabecular separation (Tb.Sp, *p* = 0.0081) and Tb.Th (*p* = 0.0204) compared with the control (Table 1). The neonatal combination of zingerone–alcohol followed by a secondary alcohol administration in adolescence (ZA + *A*) had no significant effect on the morphometry of the trabeculae (BV/TV, BS/BV. Tb. Th, Tb.Sp) compared with the control (Table 1, *p* > 0.05). Administration of alcohol in both the neonatal and adolescent stages (*A* + *A*) significantly increased the Ct.Th at the tibial midshaft compared with all treatment groups (Table 1; *p* < 0.0001). Contrarily, a significant decrease was observed in the Ct.Th of the *Z* + *W* group compared with the control (*p* = 0.0366). The combination of zingerone–alcohol during the neonatal period, followed by a second exposure to alcohol in adolescence (ZA + *A*), showed no significant effect on the Ct.Th when compared with the control (Table 1; *p* > 0.999).

**Table 1:** Micro‐CT assessment of trabecular and cortical bone in male Sprague–Dawley tibia following neonatal and adolescent treatment with zingerone (*Z*), alcohol (*A*), nutritive milk (*C*) and/or water (*W*).

	Treatment groups				
	C (*n* = 7)	*Z* + *W* (*n* = 7)	*A* + *A* (*n* = 7)	*Z* + *A* (*n* = 6)	*Z* *A* + *A* (*n* = 7)
Trabecular bone morphometry					
Proximal tibial metaphysis, voxel resolution of 14 *μ*m					
BV/TV (%)	0.97 ± 0.04	0.87 ± 0.09 ^∗∗^	0.95 ± 0.04	0.92 ± 0.03	0.97 ± 0.02
BS/BV (mm^−1^)	10.57 ± 6.67	17.32 ± 2.6 ^∗^	15.04 ± 5.03	18.71 ± 1.55 ^∗^	12.91 ± 4.75
Tb.Th (mm)	0.300 ± 0.22	0.120 ± 0.02 ^∗^	0.150 ± 0.06	0.110 ± 0.01 ^∗^	0.177 ± 0.07
Tb.N (mm^−1^)	5.00 ± 3.01	7.50 ± 0.96	7.08 ± 2.10	8.56 ± 0.51 ^∗^	6.25 ± 2.18
Tb.Sp (mm)	0.0043 ± 0.005	0.017 ± 0.013 ^∗^	0.0043 ± 0.005	0.01 ± 0.000	0.0043 ± 0.005
Total VOI (mm^3^)	29.56 ± 2.26	28.55 ± 1.22	28.06 ± 3.17	29.27 ± 3.33	26.38 ± 1.99
Cortical bone morphometry					
Tibial midshaft, voxel resolution of 29 *μ*m					
Ct.Th (mm)	3.40 ± 0.76 ^*x*^	2.23 ± 0.71 ^∗/*x*^	5.60 ± 1.14 ^∗^	2.87 ± 0.51 ^*x*^	3.04 ± 0.61 ^*x*^

*Note:* Data are presented as mean ± standard deviation. Group 1 (*C*) received no treatment in the neonatal phase and during adolescence; Group 2 (*Z* + *W*) was treated with zingerone (40 mg/kg) in the neonatal phase and water during adolescence; Group 3 (*A* + *A*) treated with alcohol during both the neonatal and adolescent phase (1 g/kg ethanol); Group 4 (*Z* + *A*) treated with zingerone in the neonatal phase and alcohol in adolescence; Group 5 (ZA + *A*) treated with a combination of zingerone–alcohol in the neonatal phase, followed by alcohol in adolescence. Nutritive milk (10 mL/kg) was used a vehicle for the administration of the treatments. *n* = 6 − 7 per treatment group. BV/TV (*%*) = bone volume fraction; *B* *S*/*B* *V* (mm^−1^) = specific bone surface density, Tb.Th (mm) = trabecular thickness; Tb.N (mm^−1^) = trabecular number; Tb.Sp (mm) = trabecular separation, and total bone volume of the proximal tibia volume‐of‐interest (mm^3^); Ct.Th (mm) = cortical thickness.

Abbreviations: A, alcohol; C, control; Total VOI, total volume of interest; W, water; Z, zingerone; ZA, zingerone–alcohol.

^∗^ *p* < 0.05 compared with the control. ^*x*^ *p* < 0.0001 compared with *A* + *A* grou*p*.

^**^ *p* ≤0.01 compared with control group

## 4. Discussion

This study investigated whether neonatal zingerone administration modulates the effects of adolescent alcohol exposure on bone health parameters and tibial microarchitecture in growing rats. Importantly, the study was not designed to assess alcohol‐induced improvements in bone parameters, but rather to examine whether zingerone confers protective or modulatory effects under conditions of alcohol exposure. In our model, alcohol administration during adolescence, with or without neonatal zingerone exposure, significantly increased BALP and/or P1NP compared with controls, whereas OC was only significantly elevated in the *Z* + *A* group compared with *Z* + *W*, but not compared with the control. Neonatal coadministration of zingerone–alcohol followed by a secondary alcohol insult during adolescence did not reduce BALP or OC levels compared with alcohol alone, but showed a moderate reduction in P1NP compared with alcohol‐only groups. Micro‐CT analysis showed that neonatal zingerone administration alone did not improve tibial microarchitecture. Alcohol exposure (*A* + *A*) was associated with an unexpected increase in Ct.Th relative to the control, a finding that was not anticipated based on the study hypothesis. The *Z* + *A* group exhibited lower Ct.Th compared with *A* + *A*; however, this is to be interpreted as a difference between treatment conditions within this experimental model and not as attenuation of defined alcohol‐induced pathological effect. Similarly, when a combination of zingerone–alcohol was administered in the neonatal phase of development (ZA + *A*), it preserved trabecular and cortical parameters compared with *Z* + *W* and *Z* + *A*, although these findings do not indicate restoration to control levels nor confirm a protective effect of zingerone. Acute alcohol dosing during both neonatal and adolescent phases increased cortical midshaft thickness compared with controls, potentially suggesting an increase in mechanical strength.

Findings showed that plasma surrogate markers of bone turnover, BALP (a marker of bone formation) and OC (a marker of bone mineralization) showed a nonsignificant upward trend, whereas P1NP (bone formation marker) was elevated when alcohol was introduced as a secondary insult (adolescent phase), with or without neonatal zingerone administration [23]. Research has shown that BALP plays a crucial role in promoting bone formation, OC contributes to the mineralization of the bone matrix, and P1NP serves as an indicator of the rate of bone turnover by reflecting the synthesis of type I collagen, a key component in maintaining early‐phase bone turnover [24, 25]. Changes in the levels of plasma biomarkers are essential indicators of bone health and turnover used in preclinical research to monitor the anabolic nature of treatments for the management of bone‐related conditions [25, 26]. Therefore, the levels of BALP, OC, and P1NP would reflect the activity of osteoblasts and the rate of bone turnover [25, 26]. Unlike chronic high‐dose ethanol exposure in adults, which is often associated with suppressed osteoblast activity and reduced BALP, OC, and P1NP, the low‐dose, intermittent ethanol exposure during periods of rapid growth in our model may have induced a transient compensatory upregulation of bone formation activity, explaining the observed increases in these markers at endpoint. However, chronic ethanol exposure has been shown to disrupt the bone remodeling process by reducing the activity of osteoblasts, causing plasma levels of BALP, OC, and P1NP to decrease [27].

In the current study, treatment with neonatal zingerone did not prevent the increase in plasma levels of BALP, OC, and P1NP induced by alcohol in the adolescent phase. The variability, particularly in P1NP, observed across groups likely reflects biological variation during rapid skeletal growth rather than a direct modulatory action of zingerone on this marker. According to Hale et al. [28], an increase in markers of bone function may not directly signify an increase in bone formation but indirectly suggest the state of bone dysfunction such as osteoporosis or Paget′s disease. The elevated plasma levels of biomarkers in our study may reflect a physiological attempt to counter oxidative stress induced by alcohol metabolism via alcohol dehydrogenase (ADH) and cytochrome P450 2E1 (CYP2E1) [29–31]. The activation of CYP2E1 accelerates the production of reactive oxygen species (ROS), which in turn overwhelms the antioxidant defense mechanisms (such as glutathione), impairing the body′s ability to neutralize ROS, leading to oxidative stress [32]. Although oxidative stress was not determined in the current study, similar models have shown that oxidative stress leads to osteoblast inhibition and bone pathology [29].

Previous research has shown that potent antioxidant and anti‐inflammatory agents such as zingerone may confer bone health benefits by modulating against the production of oxidative stress [33]. Based on Asiedu et al. [34] findings, it is evident that CYP2E1 levels were elevated in these male rat pups that were treated neonatally with alcohol and/or its combination with zingerone. Previous studies have shown that zingerone can modulate CYP2E1 expression; however, since CYP2E1 was not measured in the current study, this mechanism remains speculative. In the current study, neonatal zingerone administration did not result in a consistent normalization of plasma BALP, OC, or P1NP levels following adolescent alcohol exposure. Although differences were observed between treatment groups, these biomarker patterns cannot be clearly attributed to zingerone itself because alcohol exposure (*A* + *A*) and neonatal zingerone alone (*Z* + *W*) both produced unexpected alterations in these markers. The observed variations in BALP, OC, and P1NP therefore likely reflect a combination of developmental stage, alcohol‐driven metabolic responses, and growth‐related remodeling rather than a direct modulatory or protective effect of zingerone.

Consistent with previous studies, control rats showed normal tibial growth without significant alterations in length, mass, or BMLR [35]. However, neonatal zingerone administration followed by adolescent alcohol exposure (*Z* + *A*) led to significant reductions in tibial mass and BMLR, indicating possible structural weakening. This decrease in bone mass, linked to reduced bone formation, suggests that neonatal zingerone administration may not have been effective on its own to protect against alcohol‐induced bone impairments during adolescence [36]. Although previous studies reported no detrimental effects of zingerone on the growth and development of long‐bone indices in adult rats, our findings suggest that alcohol administration during adolescence, especially the critical developmental period involving rapid physical growth, may have caused internal structural deterioration leading to decreased bone mass [12]. However, zingerone has shown some beneficial effects in preserving longitudinal growth of the tibia when coadministered with alcohol in the neonatal phase (ZA + *A*). The nonsignificant reduction in BMLR observed in the ZA + *A* group should be interpreted with caution. Given the variability across treatment groups, it may not be possible to attribute this change specifically to zingerone, and may instead reflect combined effects of neonatal exposure and developmental or alcohol‐related influences rather than a direct protective mechanism.

Consistent with bone morphometry findings, micro‐CT analysis revealed that alcohol alone (*A* + *A*) increased Ct.Th compared with all other groups without altering trabecular parameters, aligning with studies showing site‐specific effects on cortical bone. These bone deteriorative changes are evidenced by thinner (Tb.Th), more numerous (Tb.N) and widely spaced trabeculae (Tb.Sp), with increased bone surface‐to‐volume ratios (BS/BV), indicating higher trabecular porosity and fragmentation [22]. The neonatal coadministration of zingerone–alcohol followed by subsequent alcohol administration in adolescence (ZA + *A*) had no effect on the morphometry of the trabeculae, suggesting that zingerone may possess moderate protective benefits against alcohol‐induced trabecular deterioration by preventing a significant decrease from occurring Interestingly, a double hit of alcohol, administered neonatally and in adulthood (*A* + *A*), had no effect on trabecular parameters as we had hypothesized. Although alcohol‐associated trabecular changes described in the literature were observed in *Z* + *A*  [37, 38], similar alterations were also seen in *Z* + *W*, indicating that neonatal zingerone alone may also disrupt trabecular development. The reduced Ct.Th observed in *Z* + *A* relative to *A* + *A* suggests that neonatal zingerone treatment did not prevent alcohol‐induced alterations in cortical modeling, and may reflect divergent developmental responses rather than protection.

Although classical alcohol‐induced bone loss was not observed in the *A* + *A* group, previous studies have shown that neonatal alcohol exposure can lead to variable skeletal outcomes depending on dose, duration, and developmental timing [39]. Our findings may reflect a transient compensatory response or limitations of the current experimental conditions, and they underscore the need to interpret the potential or relative effects of zingerone within this context. Although multiple technical replicates and region‐specific assessments were performed to strengthen the reliability of our findings, these do not substitute for biological replication. Therefore, future studies with larger cohorts should be employed for further evaluation.

Despite the well documented pharmacological benefits of zingerone, including its antioxidant, anti‐inflammatory, antimicrobial, anticancer and radioprotective properties, its effects in this study were modest and inconsistent across bone markers and morphology, depending on whether it was administered alone or with alcohol [40, 41]. Future studies should consider introducing a secondary insult of zingerone in the adolescent phase to further investigate its potential role in modulating bone health later on in adulthood.

The alcohol model in this study, especially when introduced later on in adolescents, induced a significant effect on plasma markers of bone function as well as bone morphometry and morphology parameters. Rats receiving alcohol during both critical periods of developmental plasticity (*A* + *A*) exhibited thicker cortical bone and unchanged trabeculae at the proximal metaphysis, aligning with findings by Gaddini et al. that low‐dose alcohol and specific drinking patterns may have site‐specific benefits on bone health, particularly by enhancing the structural integrity of cortical bone [42, 43]. In our experimental model, low doses of alcohol (1 g/kg) administered to rat pups mimic indirect alcohol consumption by infants from breastfeeding mothers consuming one to two beverages/day [44]. This has been shown to be enough to reduce hip and spine BMD in humans [45, 46]. However, findings from the current study suggest that acute‐low doses of alcohol may benefit bone density without necessarily improving mechanical strength. These findings may reflect a dysregulation of bone remodeling, as alcohol has been extensively documented to disrupt normal bone metabolism, which could explain the observed changes in Ct.Th and P1NP levels [9]. Clayton et al. [47], reported that chronic alcohol consumption can reduce mechanical strength independently of BMD, highlighting the complex dose‐, duration‐, and pattern‐dependent variability of alcohol on bone health [43, 45, 48]. This discrepancy underscores the need to consider not only structural bone parameters but also biomechanical properties and remodeling dynamics when evaluating the effects of alcohol on skeletal integrity.

Zingerone has been reported to exhibit positive bone health benefits by slowing osteoclast‐driven bone resorption; however, it alone does not adequately support bone homeostasis or prevent long‐term structural deterioration. Its natural origin and low‐toxicity profile make it a promising candidate for future research, especially in combination with other bone anabolic agents to enhance bone regeneration and health.

## 5. Conclusion

This study provides preliminary insights into how neonatal zingerone exposure, alone or combined with alcohol, interacts with adolescent alcohol intake to influence skeletal development. Within the limitations of a small sample size and the modest effect sizes, our findings show that alcohol exposure during development produced complex, site‐specific changes in bone, including increases in Ct.Th and P1NP and preservation of trabecular bone volume, reflecting altered or dysregulated bone modeling rather than classical bone loss. Neonatal zingerone alone showed little evidence of bone protection, whereas its coadministration with alcohol modestly attenuated some alcohol‐associated structural changes, though effects were inconsistent across bone compartments. These findings emphasize the importance of optimizing both developmental timing and dosage in interventions designed to target bone health. The moderate efficacy of zingerone as a standalone bone anabolic agent suggests potential for its use in combination therapies, but further research is needed to clarify its bone mechanism and optimize its application for bone‐related conditions. Future studies with larger sample sizes, broader bone health assessments (i.e., ovariectomized murine models) and longer intervention periods are warranted to clarify whether zingerone, especially when used as a dietary supplement, has clinically meaningful bone‐protective effects, either alone or part of combination therapies.

## Author Contributions

B.D.V., T.T.N., A.E.K., and A.M.J. contributed to the conceptualization of the study. Data curation was performed by B.D.V. and T.T.N. Funding acquisition was managed by A.M.J. Methodological assistance was provided by B.A. All coauthors participated in the review and editing of the manuscript.

## Funding

This study was supported by the National Research Foundation (10.13039/501100001321) (121828).

## Ethics Statement

This preclinical study was ethically approved at the University of Pretoria by the Faculty of Health Sciences Research Ethics Committee (Ethics Number 491/2022). The exclusive use of male rats minimized the confounding effects of sex hormones, allowing for a more focused investigation of alcohol′s independent effects on bone development and metabolism.

## Conflicts of Interest

The authors declare no conflicts of interest.

## Supporting information


**Supporting Information** Additional supporting information can be found online in the Supporting Information section. Figure S1: This figure provides an overview of the micro‐CT workflow, including sample preparation, imaging setup, equipment details, image reconstruction, and analysis for tibial morphometry. Figure S2: This figure provides an illustration of the extracted sections from tibia samples used for micro‐CT analysis, highlighting voxel sizes for trabecular bone density and cortical thickness measurements.

## Data Availability

The data that support the findings of this study are available from the corresponding author upon reasonable request.
