# Supplementary material for: The Effects of Neonatal Zingerone Administration and Adolescent Alcohol Exposure on Bone Health Markers and Morphometry in Male Sprague–Dawley Rats
Source: Biomed Res Int. 2026 Apr 29;2026:7060593. doi: 10.1155/bmri/7060593 (PMC13128980; doi:10.1155/bmri/7060593)
Supplement: Supplementary file 1 — Supporting Information Additional supporting information can be found online in the Supporting Information section. Figure S1: This figure provides an overview of the micro‐CT workflow, including sample preparation, imaging setup, equipment details, image reconstruction, and analysis for tibial morphometry. Figure S2: This figure provides an illustration of the extracted sections from tibia samples used for micro‐CT analysis, highlighting voxel sizes for trabecular bone density and cortical thickness measurements. [file BMRI-2026-7060593-s001.docx]

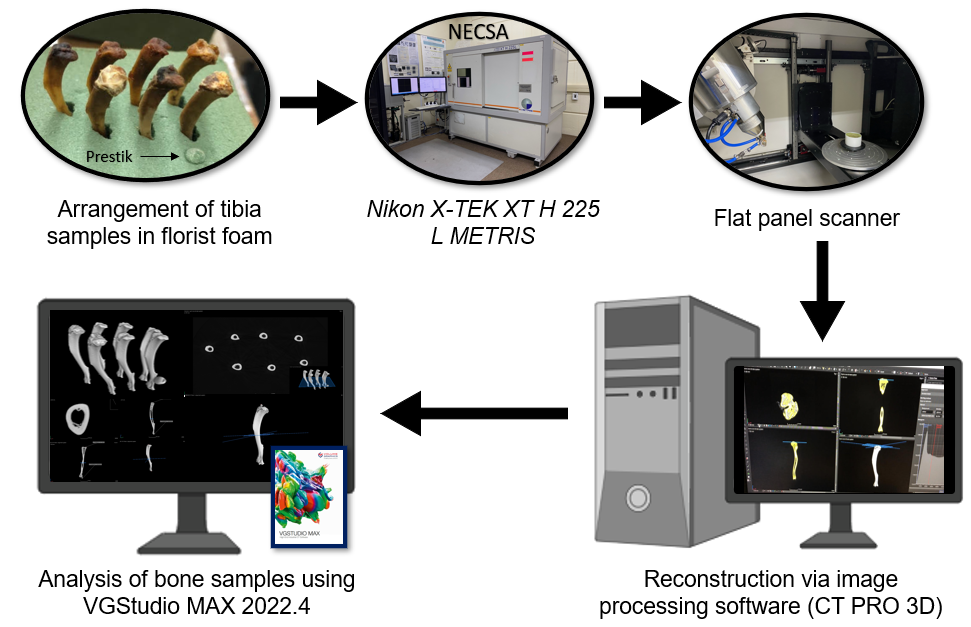


Figure S1: Micro-CT workflow. (A) Sample preparation and imaging setup using florist foam and Prestik. (B) Micro-CT instrument (Nikon X-TEK XT H 225 L Metris, NECSA, Tring, United Kingdom) lead cabinet and PC. (C) Flat panel detector (Perkin Elmer, 16-bit dynamic range, 400 × 400 mm in size with a pixel size of 200 × 200 microns). (D) Reconstruction of images using reconstruction software (Nikon CT Pro 3D^®^). (E) Analysis of images were performed to obtain quantitative information on the morphometry of the tibiae (VGStudio MAX 2022.4 software).


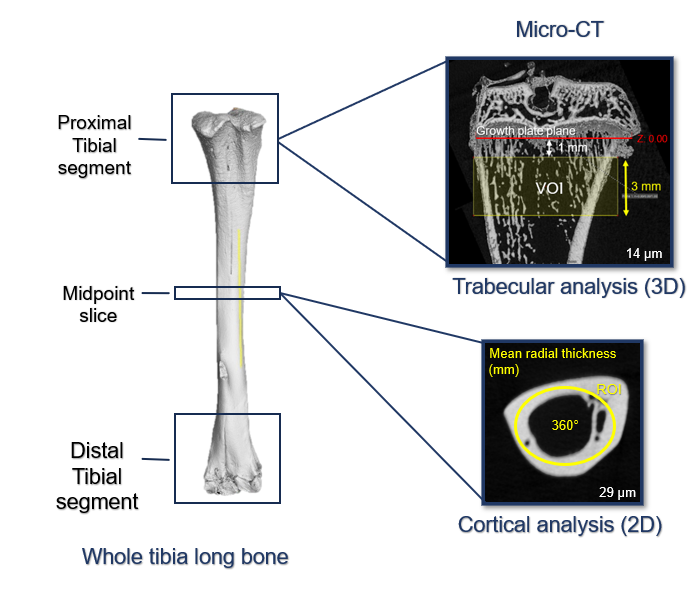


Figure S2: Illustration of the sections (3D VOI and 2D ROI) that were extracted from the tibia long bone samples for the analysis of bone microarchitecture using micro-CT imaging. The proximal tibial segment of the tibia was scanned at a voxel size of 14 µm for in-depth trabecular bone density evaluations. Whole bones were scanned at a voxel size of 29 µm to obtain the midpoint slice as a measure of cortical bone thickness. 2D: two-dimensional; 3D: three-dimensional; Micro-CT: microcomputed tomography.
